# Novel empirical models & comparative probabilistic analysis of interconnectedness of volcano eruption & nearby earthquakes

**DOI:** 10.1371/journal.pone.0320210

**Published:** 2025-04-16

**Authors:** Debashis chatterjee, Amlan Banerjee, Shiladri Shekhar Das, Prithwish Ghosh

**Affiliations:** 1 Department of Statistics, Visva Bharati, Santiniketan, India; 2 Geological Studies Unit, Indian Statistical Institute, Kolkata, West Bengal, India; 3 Department of Statistics, North Carolina State University, Raleigh, North Carolina, United States of America; National Autonomous University of Mexico Institute of Geophysics: Universidad Nacional Autonoma de Mexico Instituto de Geofisica, MEXICO

## Abstract

This paper takes a probabilistic approach to validate novel empirical models and the directional distributional similarities of nearby earthquake counts related to a typical volcano with its eruption duration. Considering the datasets on volcanic eruptions and earthquakes, focusing mainly on earthquakes within a 100-kilometer radius and within a three-year time frame of the volcano eruption. An empirical probabilistic models for the same; statistical model validation tests favor the proposed models is proposed. In addition, a novel directional statistical approach to characterize the interconnection and distributional similarities of volcanic eruptions and earthquakes near volcanoes, utilizing the directional nature of the datasets. The project and partition the volcanic eruption and earthquake data to assess its directional distribution is shown. The analysis demonstrated that the data adhered to a Von Mises distribution and unsupervised equal partition revealed for both datasets, highlighting the interconnected nature of volcanic eruptions and earthquakes. Also, Von Mises-Fisher distribution fit test is applied to this work; the analysis produced partition results that closely aligned with the partitions obtained through the 2D projection. This congruence emphasizes the robustness of the findings in a spherical context. the proposed empirical models and conclusions on distributional similarities may provide insights into the underlying mechanisms connecting these geological phenomena.

## 1 Introduction

Accurately assessing volcanic hazards is the primary step in preventing disasters [1]. Most earthquakes emerge along the edges of tectonic plates, where most volcanoes are situated. However, at least theoretically, not all earthquakes can be related to volcanoes. The plates’ interaction, not magma’s movement, causes most earthquakes. On the other hand, the movement of magma also causes most earthquakes near a volcano [2,3]. Deep volcanic tremor and magma ascent mechanism case study leading to an earthquake is addressed in many literatures like [4–7]. Once the volcano is open and magma flows through it, constant earthquake waves, called harmonic tremors, are recorded [2,7].

Earth Science-related literature on the relationship between volcano eruption and nearby occurring earthquakes is plenty. A review of how earthquakes trigger volcanic eruptions has been addressed in [7]. [8] gathered observations concerning how mud volcanoes and various geological systems (including earthquakes, volcanoes, liquefaction, groundwater, and geysers) respond to seismic activity. The collected data of [8] reveals a distinct threshold of distance (100 KM)[9] that triggers the responses. The categorization developed in [7] demonstrates that most can be triggered by seismic activity, although they necessitate specific combinations of volcanic and seismic conditions [10]. Triggering is improbable unless the volcanic system is in a state primed for eruption. Seismically-induced unrest is more prevalent, especially in connection with hydrothermal systems. Interactions between earthquakes and volcano activity are statistically addressed in [2,11]. [12] addressed Changes over time in the characteristics of shallow volcano-tectonic earthquakes linked to the escalation of volcanic activity at the Kuchinoerabujima volcano, Japan. Researchers are actively developing statistical methods and diverse models within the time-space-magnitude parameter space of earthquakes and advances in pursuit of suitable stochastic modeling techniques that rely on the history of earthquake occurrences and pertinent geophysical information. This endeavor aims to describe and forecast earthquake activity accurately. These advancements aim to analyze seismic activity using regularly accumulated earthquake hypocenter catalogs, e.g., see [13], [14]. In particular, [14] reported a truncated exponential frequency-magnitude relationship observed in earthquake statistics. [15] addressed earthquake statistics and its importance. A statistical model is designed to characterize ground motion generated by earthquakes at local and regional distances [16]. Recent model-based earth science research is adapting directional statistical tools. For instance, scaled von Mises–fisher distributions and regression models for paleomagnetic directional data have been proposed in [17]. The probabilistic assessment of volcanic hazards made in [18] serves to quantify volcanic hazards and elucidate uncertainties about the magnitude and potential consequences of volcanic activity. In particular, [18] presents an approach developed to estimate volcanic hazards related to tephra fallout and illustrates this approach with a tephra fallout hazard assessment for the city of Leon, Nicaragua, and the surrounding area. [19] proposed a Bayesian approach for the determination and parameterization of earthquake focal mechanisms. [20] demonstrated a numerical simulation of earthquake-induced excitation on tire-reinforced sand behind a retaining wall. [21] addressed elastoplastic models for pressure sources in a heterogeneous domain were developed to characterize, evaluate, and interpret observed deformation in volcanic regions and used the Finite Element Method (FEM) to simulate deformation in a three-dimensional domain, partitioned to incorporate volcano topography and the distribution of heterogeneous material properties.

In the discussion of [22] provides an overview of Probabilistic Tsunami Hazard Analyses (PTHAs), highlighting the challenges of assessing diverse tsunami sources, modeling their impacts, and estimating hazards while accounting for uncertainties to inform risk reduction efforts globally [23]. This study [24] introduces a novel monitoring approach for natural hazards using Hidden Markov models (HMMs) with Poisson emissions, leveraging an empirical recurrence rates ratio (ERRR) to enhance risk forecasting and state predictions, demonstrated through analyses of Hawaiian volcanic and West Atlantic hurricane interactions [25]. This study [26] examines the resilience of physical-logical interdependent networks to seismic events, using a novel Localized Attacks with Probabilistic Failures (LAPF) model to show that seismic attacks can cause more catastrophic failures than traditional localized attacks, and emphasizes the importance of protecting critical logical bridge nodes to enhance network robustness [27].

Based on novel measurements, statistical estimations, and models, [2] supported the speculation that a large earthquake can trigger subsequent volcanic eruptions over surprisingly long distances and time scales. However, the stress changes from usual earthquakes are typically smaller than those associated with solid-earth tides (about 0.001 MPa) and cannot directly influence a typical volcano eruption [2,5]. [28] investigated the interaction between earthquakes and volcanic eruptions by analyzing a modern seismic data catalog and eruption records. The conclusion of [28] is that moderate earthquakes having moment magnitudes of 5 to 6 (Mw 5 to 6) are triggered within a 50 km horizontal distance from volcanoes for approximately 0.3 years after the initiation of an eruption and roughly 13% of volcanic eruptions are escorted by medium earthquakes. The likelihood of quakes every 0.1 years for 0.3 years after an explosion is about five times larger than regular. There are many natural cases, such as a recent example of earthquake-mud volcano triggering that surfaced when a mud volcano on the island of Bharatang, on the island of the Middle Andaman [29].

### 1.1 Objective & overview of this paper

1. Holistically, the volcano, and nearby earthquake distribution fall under a directional statistical paradigm because of the inherent directional parameters such as latitude and longitude involved in the data. To the knowledge, little literature exists that proposes a novel regression model capturing the inter-dependencies of volcanoes and nearby earthquakes and uses directional statistical models to frame the research question on the inter-relationship of earthquakes and nearby volcanoes and proceed toward answers. In addition, The application the Von mises-fisher clustering models presented in [30] on Volcano eruptions and their nearby earthquake occurrences and address the goodness-of-fit. It is proposed and validated a novel empirical model of volcanoes and their nearby earthquakes. Then performing a comparative statistical analysis of directional earthquakes and volcanic eruptions. Probabilistically searched for the geological directional relationship between volcanic eruptions and earthquakes by creating a secondary dataset that considers location and time factors. A empirical statistical models and explore directional distributional properties and similarities among the occurrence of volcano eruption and its nearby earthquake numbers and magnitude is proposed.
2. Theoretically, there should be an abundance of observed earthquakes before a volcanic eruption and vice versa [7]. In this paper, A proposed empirical statistical models and wish to apply directional statistical tools to verify the same is introduced [31].
3. The proposed empirical statistical models to express the interconnecting nature of volcanic eruptions and earthquakes. Data were collected on volcanic eruptions and earthquakes, focusing on events occurring within a 100-kilometer radius and within a one-year time-frame. By examining the count data for the occurrences of these natural phenomena, it is intend to uncover and quantify the distributional similarities and employ directional statistical approaches.
4. In addition, combined volcanic eruption and earthquake data were projected and partitioned to assess its distribution.
5. Application of the Von Mises-Fisher distribution fit test to delve deeper into the spherical domain. This analysis yielded partition results that closely aligned with the partitions obtained through the 2D projection. This congruence underscores the robustness of the findings in a spherical context.

## 2 The dataset

### 2.1 About the earthquake and volcano irruption dataset

**Fig 1:**
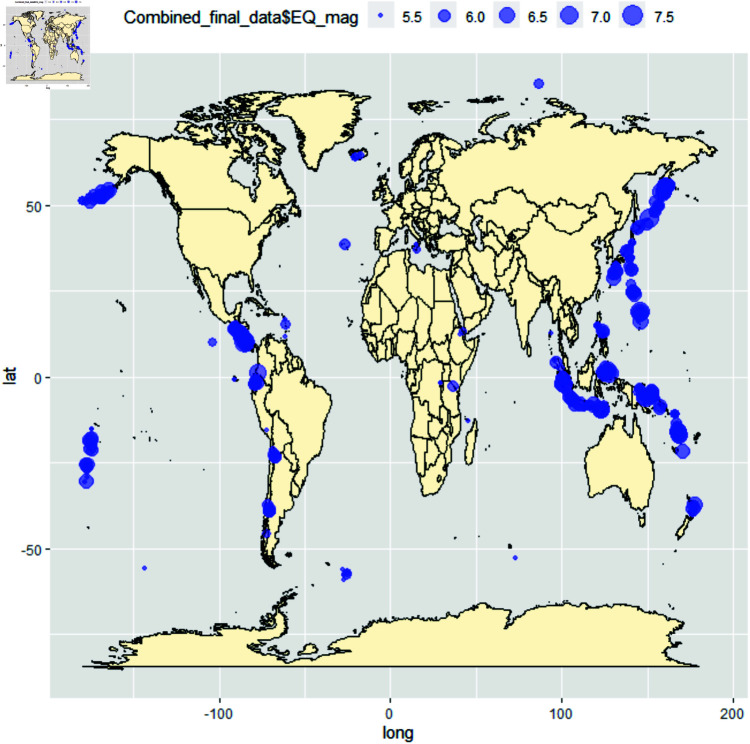
Plot of the recorded earth quack observed worldwide concerning the earthquake magnitude. Here, the point sizes will increase concerning the magnitude. The bigger magnitude means a bigger plot of that dataset.

**Fig 2:**
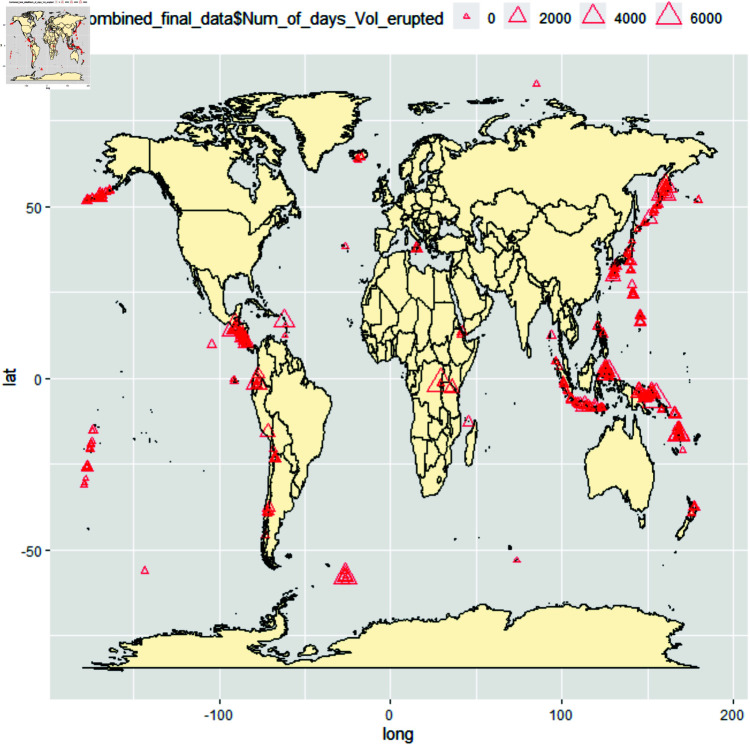
Plot of the recorded volcano eruption all over the world map concerning the number of days volcano eruption happened. The larger the triangle is, the larger the volcanic eruption time as per the dataset.

The National Earthquake Information Center (NEIC) is tasked with identifying the precise locations and magnitudes of noteworthy earthquakes across the globe and sharing this information with scientists and scientific organizations. This database is established through advanced national and global seismograph networks and collaborative international agreements. The NEIC earthquake dataset is collected from https://www.usgs.gov/programs/earthquake-hazards/earthquakes, given in Figs 1 and 2. The second dataset provideswith the volcano eruption; the volcanic dataset is collected from https://volcano.si.edu/, and the view of the volcano eruption is given in Fig 2. Both data sets are uploaded to the Harvard dataverse doi: 10.7910/DVN/SPLMMN.

### 2.2 About the novel secondary data set creation

Considering the two datasets about the volcanic eruption and the earthquake information. Initially, The earthquake data set had 37331 data points, while the Volcano data set had 1193 data points.

Then a hypothetical manual threshold of 100 KM around the volcanic eruption and sorted and found out how many earthquakes happened within that threshold.

Then, for the time points, it is considered an another threshold which tells us, considering the previous threshold, how many earthquakes happen within the time point 365 days or one year.

Considering those thresholds, with the earthquake and volcanic dataset, which occurs within the 100 km radius and within the time point of 1 year. This gavethe final combined dataset of 478 data points and 16 parameters, givinginformation about the volcano Eruption and Earthquake.

It is hypothesize that earthquakes can trigger volcanic eruptions, and volcanic eruptions can also trigger earthquakes. Then the data is partitioned into two parts: one is the volcano that triggered the earthquake, and another one is the earthquake triggered by the volcano, which led to two datasets, one with 215 data points and 16 parameters and another one with 263 data points with 16 parameters.

The pictorial illustration of the secondary dataset creation is given in Fig 6 as an example, where it is exhibited for one exemplar Volcanic Eruption.

## 3 Methodologies

### 3.1 Novel empirical models on earthquake count & volcano eruption time

**Fig 3:**
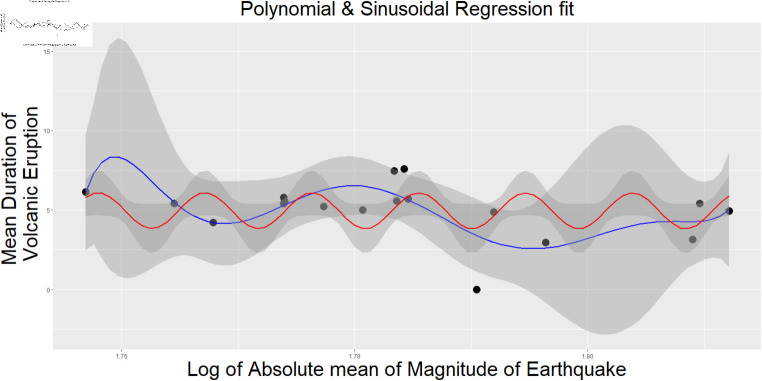
Plot of the absolute mean magnitude of earthquake vs mean period of volcano eruption. The use of the absolute mean of the earthquake’s magnitude nearby vs the mean of the volcano’s duration for the observed earthquake count near the volcano. The threshold distance is 100 km and three years. The polynomial regression is used with 7 degrees to plot(for 5 degree polynomial regression fit, *R*^2^ is 0.341).

**Fig 4:**
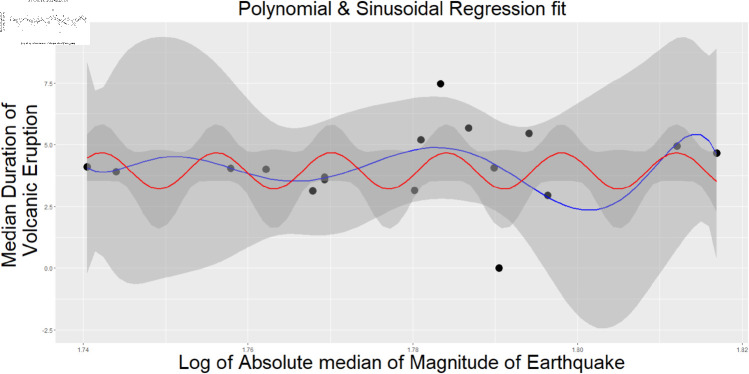
The use of the median of the earthquake’s magnitude nearby vs the Median of the volcano’s duration is used as per the dataset we created. The threshold distance is 100 km and three years. The multiple *R*^2^ is 0.1597. Then it is used the polynomial regression with 7 degrees to plot( polynomial regression fit, *R*^2^ is 0.159).

In Figs 3 and 4 we illustrate the absolute mean magnitude of the earthquake vs. the mean period of volcano eruption & The use of the Median of the Earthquake’s Magnitude nearby with respect to the Median of the Volcano’s Duration is used as per the dataset we created.

**Fig 5:**
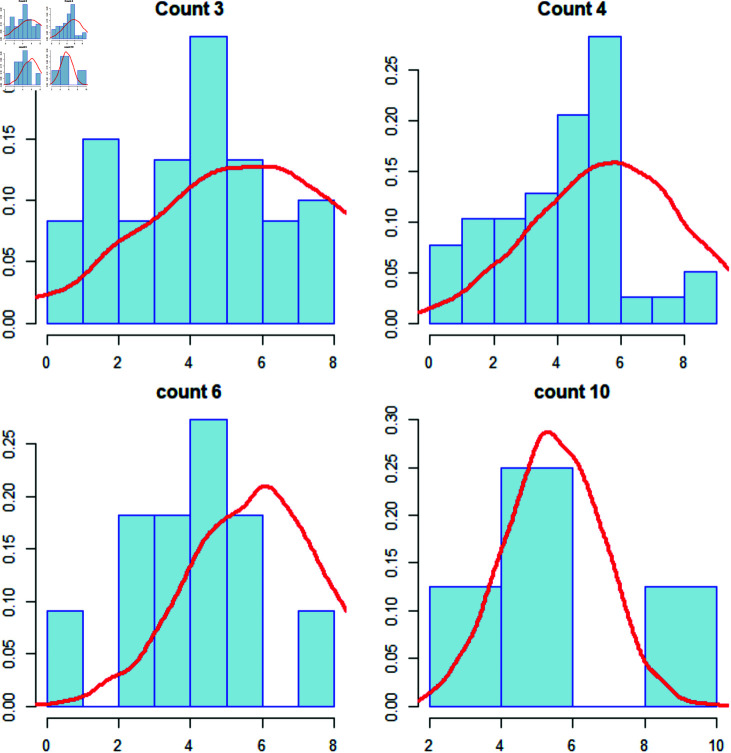
Plot of volcanic eruption duration(logarithmic transformation) for counts of earthquake happened within 100 km and three years of the volcano. Here, it is considered that the earthquake count of 3, 4, 6 and 10. here, the red line indicates the sample drawn from normal distribution from the proposed model given in the model 1. It is statistically verify that the model 1 assumption is valid for all the cases (Table 1). see Remark 4 for detailed explanation.

A simple visualization of the logarithm of volcanic eruption time vs. nearby (100 km range) earthquake count over the past three years (Fig 5) reveals a notable pattern, suggesting a noteworthy probabilistic theoretical association. The following observations are apparent:

1. The Mean duration of the logarithm of volcano eruption time seems sinusoidally increasing with the number of earthquakes observed before eruption starts.
2. The precision in determining the duration of volcano eruption increases as the number of earthquakes observed increases.

Defined two random variables: the volcanic eruption time (days) is denoted by *D*, and the nearby (100 km range) earthquake magnitudes over the past three years as *M_i,Ni_* (*N_i_* denote the corresponding earthquake count) for the *i* the volcano. If *N* many volcanoes in the dataset, it is denoted by $D=\{D_{1},D_{2},\cdots \;,D_{N}\}$. For each *D_i_*, suppose having $M_{i}:=\{M_{i,1},M_{i,2},\cdots \;,M_{i,N_{i}}\}$ many earthquakes count over the past three years (100 km range), which is denoted as *N_i_*.

1. A(log ⁡ Mi):=1Ni ∑ ⁡ k=1Ni log ⁡ Mi,k (log mean),
2. B(log ⁡ Mi):=median {log ⁡ Mi,1,log ⁡ Mi,2,⋯,log ⁡ Mi,Ni} (log median).

**Model 1 (1st empirical Model on Volcano eruption Time**). *Consider the conditional random variable*  [log ⁡ Di|Mi]. *It is hypothesized that *


E(log ⁡ Di|Mi,Ni)=p(A(Mi)), [log ⁡ Di|Mi,Ni]∼N (p(A(Mi)),σ2Ni1.35),

*and similarly,*


E(log ⁡ Di|Mi,Ni)=p(B(Mi)), [log ⁡ Di|Mi,Ni]∼N (p(B(Mi)),σ2Ni1.35),

*Where* *p* ( ⋅ )  *is a polynomial of the unknown degree to be determined using the goodness of fit,* *N* ( ⋅ )  *denotes normal distribution, and* *σ* *is an unknown model parameter to be estimated using MLE (Maximum likelihood estimation).*

**Model 2 (2nd empirical Model on Volcano eruption Time**). *This model is the same as model 1, except the mean is hypothesized to be a sinusoidal function. Consider the conditional random variable*  [log ⁡ Di|Mi,Ni]. *It is hypothesized that*


E(log ⁡ Di|Mi)=asin ⁡  (b (A(Mi)+c))+d, [log ⁡ Di|Mi,Ni]∼N (p(asin ⁡  (b (A(Mi)+c))+d,σ2Ni1.35),

*and similarly,*


E(log ⁡ Di|Mi)=asin ⁡  (b (B(Mi)+c))+d, [log ⁡ Di|Mi,Ni]∼N (p(asin ⁡  (b (B(Mi)+c))+d,σ2Ni1.35),

*where* *a,b,c,d,α,β,σ* *are unknown model parameters. It is estimated using MLE (Maximum likelihood estimation).*

**Remark 1.** *In the empirical models 1 and 2. The factor* $\frac{\sigma^{2}}{N_{i}^{1.35}}$ *is empirical (based on observation). Had it been replaced by an unknown parameter,* 1.35 *is a close approximation to the maximum likelihood estimate (MLE) of that parameter.*

**Remark 2.** *Done goodness-of-fit for polynomial regression. It is obtained that the best degree for polynomial* *p*(*M_i_*) *is 7 (0.341 is the multiple* *R*^2^ *Value) and 7 (0.1597 is the multiple* *R*^2^ *Value) for the Absolute mean and Median of volcanic eruption duration, respectively.*

**Model 3.** *Here, it is hypothesized that the joint distribution of the logarithm of the mean of the Earthquake’s magnitude nearby and the* *i^th^* *Volcano’s Duration* *D_i_*,  ( *i* ∈ { 1 , 2 , ⋯ , *N* }  *follows a bivariate normal distribution. The threshold distance is 100 km and three years. For convenience of notation, may denote the sample points with* (*x_i_,y_i_*) *defined as (mean of Earthquake Magnitude, Volcano Duration).*


[log ⁡ Di,log ⁡ A(Mi)]∼ϕ (log ⁡ Di,log ⁡ A(Mi)), (1)

*where bivariate normal density* $\phi (x,y)=\frac{1}{2\pi |\Sigma {|}^{1∕2}}\exp\left(-\frac{1}{2}{\left(x-\mu \right)}^{T}\Sigma^{-1}(x-\mu )\right).$ *Here* μ, Σ=$\left[\begin{array}{cc} \sigma_{x}^{2} & \rho \sigma_{x}\sigma_{y}\\ \rho \sigma_{x}\sigma_{y} & \sigma_{y}^{2} \end{array}\right]$ *are unknown parameters.*

**Remark 3.** *Then obtained the estimated variance is* *σ_x_*=0.0985,*σ_y_*=3.75*, and the correlation* *ρ*=0.00901*. The two parameters are taken with logarithmic transformation. Fitting Bivariate normal distribution (see [32]). Then used the Anderson Darling and Cramer Von Mises test [33] for the goodness of Fit, yielding results in the hypothesis. For Anderson- Darling test [34], which gives the p-value = 9.999e*^−5^ *and for the Cramer Von Mises test gives the p-value = 9.999e*^−5^.

The estimated mean vector and covariance matrix can then be used to characterize the fitted bivariate normal distribution.

**Remark 4.** *The KS test (Kolmogorov–Smirnov test, see [35]) is applied for model validation of the proposed empirical model 1. The p-values of the count are in Table 1, which asserts the model 1 assumption satisfies all the cases. Note that Model 2 is very similar to model 1 (same variance assumption).*

**Table 1:** Kolmogorov–Smirnov-test for every count with respect to the model 1, from where the men and variances are estimated

Count	P-values	Count	P-values	Count	P-Value
1	2.2e-16	6	0.1438	11	0.5502
2	1.039e-06	7	0.001103	12	0.2962
3	0.1697	8	0.1058	13	0.1798
4	0.5475	9	0.05988	14	0.8619
5	0.1123	10	0.05257	15	0.0774
16	0.2186	17	0.2322	18	0.9745

Fig 5 is the density Plot of the Duration of volcanic eruption concerning the count of Earthquakes observed within a 100 km radius and three years of the volcano. Here, it is considered the earthquake count of 3,4,6 and 10. Here, the red line indicates the sample drawn from normal distribution from the proposed model given in the model 1. Fig 3 shows the fitted regression line for the 5-degree regression model where the absolute mean is used for the Earthquake’s Magnitude nearby vs. the mean of the Volcano’s Duration for the observed Earthquake count near the Volcano. The threshold distance is 100 km and three years, and Fig 4 shows the regression model of a 10-degree polynomial where the Median of the Earthquake’s Magnitude nearby vs the Median of the Volcano’s Duration is used, as per shown in Fig 2. The threshold distance is 100 km and three years. The multiple *R*^2^ is 0.3649. The detailed table for all polynomial regressions for both, starting from 1 degree to 10 degrees, is given in the supplementary materials. Fig 7 shows the Plot of the Count dataset where the arithmetic mean of the Magnitude of the Earthquake vs the Duration of the Volcano and overlayed the Contour plot of Bivariate Normal Distribution is used.

**Fig 6:**
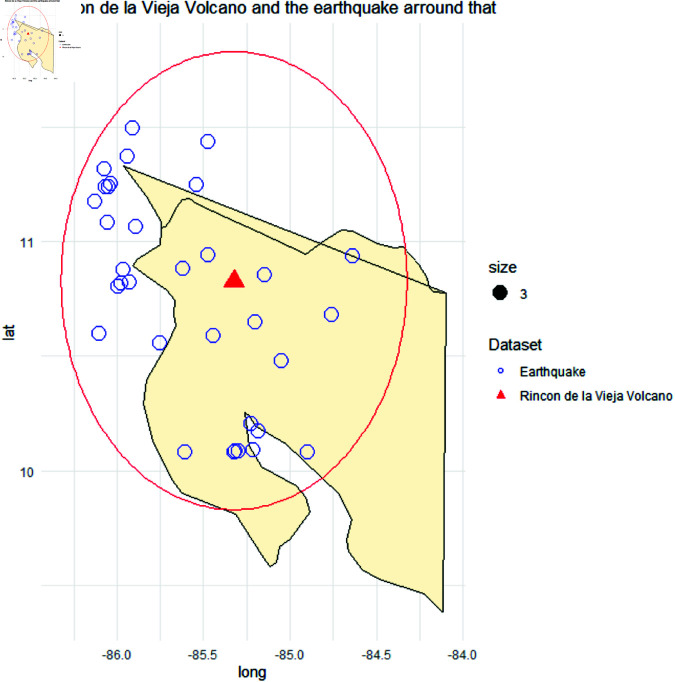
This plot illustrates one instance of creating the dataset. Then plotted the location (latitude, longitude) of 1 typical volcano (Rincon De Vieja), indicated by the Red Triangle, and the earthquake that happened near its 100 km (denoted with the blue circles) that happened in the coastal area of U.S.

**Fig 7:**
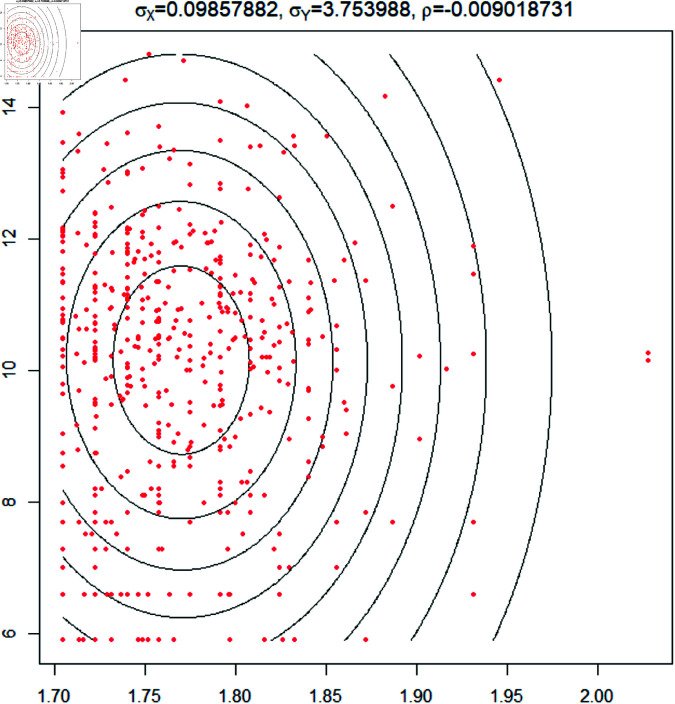
Plot of the arithmetic mean magnitude of earthquake vs period of volcano eruption. The arithmetic mean of the earthquake’s magnitude nearby vs the volcano’s duration is used. The threshold distance is 100 km and three years. Here (x, y) is defined as (arithmetic mean of earthquake magnitude, volcano duration). Here the estimated variance is *σ_x_* = 0.0985, and *σ_y_* = 3.75 and the covariance *ρ* is 0.00901. The two parameters are taken with logarithmic transformation. Fitting Bivariate normal distribution, used the Anderson- Darling test, which gives the p-value = 9.999*e*^−5^ and the Cramer Von Mises test gives the same p-value = 9.999*e*^−5^.

### 3.2 A spherical Mixture model based Statistical Approach

Formally, a mixture model corresponds to the mixture distribution representing the probability distribution of observations in the overall population. The Gaussian mixture model is commonly extended to fit a vector of unknown parameters (here, Von Mises Fisher Distribution) [36,37].

Then defined the density for directional parametric mixture distribution *p* ( *x* | *𝜃* ) , where $𝜃=({𝜃}_{1},{𝜃}_{2},\cdots \;,{𝜃}_{K})$ represent appropriate parameter set, as follows.


$$\begin{array}{ccc} p(𝜃)=\overset{K}{\underset{i=1}{\sum }}\phi_{i}F(x|{𝜃}_{i}). & & \end{array}$$ (2)

In the previous article [37,38] it is clearly mentioned all the parameters and necessary points. For fitting the mixture Von Mises Fisher distribution to both datasets, Use of the help of R package **movMF** [39,40].

## 4 Results

### 4.1 Directional statistical results

Refer to [41] and [42] for essential definitions related to directional statistics. Moreover, some essential definitions and properties of certain directional statistical distributions and tests in the Supplementary file. Circular data analysis techniques are employed to study the characteristics of observed Earthquake locations, which inherently exhibit cyclical or angular properties. In earthquake scenarios, these locations are often measured in degrees along a circular scale, mirroring the Earth’s surface.

### 4.2 Results on projected circular distributions of earthquake & volcano on theearth

Observe that the spherical plot of the raw data (Figs 1 and 2) indicates clusters and the need for partition. Indeed, for unpartitioned whole data, Table 2 says the test rejects the null hypothesis for the Watson Test for Dataset of Earth Quake for 0.01 level of Significance both of location parameters, so overall unpartitioned locations of the earthquake as a whole does not follow the Von Mises distribution. Indeed, it is shown that both data indicate the same number of partitions under unsupervised learning. The results of Homogeneity for the whole dataset are mentioned in Table 3, which tells that if not partitioned, the entire dataset of the two location parameters rejects the null hypothesis.

**Remark 5.** *Tables 2 and 3 and Table 3 indicate that the location parameter as a whole does not follow the proposed circular distribution (von Mises), and the spherical plot of the raw data plot of Figs 1 and 2 indicates clusters and need for partition. Hence, the immediate logical conclusion is partitioning the whole dataset and testing for spherical distributional similarity.*

**Table 2:** Watson Test Result for Dataset of Volcano Eruption and Earthquake dataset for 0.01 level of Significance both of location parameters of the dataset does not follow a von Mises distribution.

Earthquake			
	Parameter	Test Statistics	Critical value
	Longitude	0.237	0.081
	Latitude	0.0454	0.09
**Volcano**			
	Longitude	0.3133	0.081
	Latitude	0.1395	0.09

**Table 3:** Test of Homogeneity to get if both the location parameters of the Volcano Eruption and Earthquake dataset follow the same distribution or not.

	Test statistics	Critical Value	Output of null Hypothesis
**Earthquake**	0.318	0.268	Reject Null Hypothesis
**Volcano**	0.6433	0.268	Reject Null Hypothesis

**Table 4:** Hypothesis test for von Mises-Fisher distribution over Kent distribution for earthquake dataset where the p-value is 0.583 and the null hypothesis is whether a von Mises-Fisher distribution fits the data well, where the alternative is that Kent distribution is more suitable and similar for volcano dataset where the p-value of this dataset is 0.5270.

Data part	Test	Bootstrap p-value
Earthquake	195.979	0.583
Volcano	194.7918	0.5270

### 4.3 A comparison between Kent distribution and Von Mises distribution

In this section, the limiting null distribution of Kent’s statistic is used (see [43]) to test whether a sample comes from the Fisher distribution (Von Mises Fisher distribution) when K, the concentration parameter, goes to *inf* ⁡ . A modification is suggested, the limiting null distribution of which is $\chi_{2}^{2}$ when either *κ* or n, the sample size, goes to *⁡*. Tests of Fisher based on the eigenvalue of the sample cross-product matrix are also considered. Numerical examples are presented [44].

Here testing of the Hypothesis test for von Mises-Fisher distribution over Kent distribution for earthquake and volcano datasets. The null hypothesis is whether a von Mises-Fisher distribution fits the data well, whereas the alternative is that the Kent distribution is more suitable. The details of the hypothesis testing with the p-value are given in Table 4.

### 4.4 Results for two-dimensional partition of the dataset

Table 5 contains the partition of the combined earthquake and volcano dataset, which givesseven partitions which are following vonMises distribution, considering the projection of the location parameter and Individually tested the distributions of that dataset with the help of Watson Test.

**Table 5:** This table contains the partition of the combined earthquake and volcano dataset, which givesseven partitions that follow Vonmises distribution considering the projection of the location parameter and Individually tested the distributions of that dataset with the help of Watson Test.

EQ Lat	EQ Long	Vol Lat	Vol Long	Dist
–56.254 to 55.918	–177.64 to 177.26	–56.300 to 56.056	–177.19 to 177.18	Von Mises
–37.341 to 85.644	–177.16 to 177.33	–37.520 to 85.608	–177.19 to 177.18	Von Mises
–39.744 to 56.170	–177.98 to 177.52	–39.420 to 56.056	–177.19 to 177.18	Von Mises
–59.307 to 64.681	–179.95 to 176.17	–59.017 to 64.633	–179.03 to 179.58	Von Mises
–30.199 to 55.918	–178.09 to 168.46	–30.543 to 56.056	–178.56 to 168.37	Von Mises
–57.679 to 64.782	–178.36 to 177.33	–57.80 to 64.42	–177.17 to 177.18	Von Mises
–57.241 to 63.965	–90.91 to 158.93	–57.800 to 63.983	–90.60 to 158.20	Von Mises

### 4.5 Results regarding the fit of Mixture of Fisher Von Mises DistributionModel

**Fig 8:**
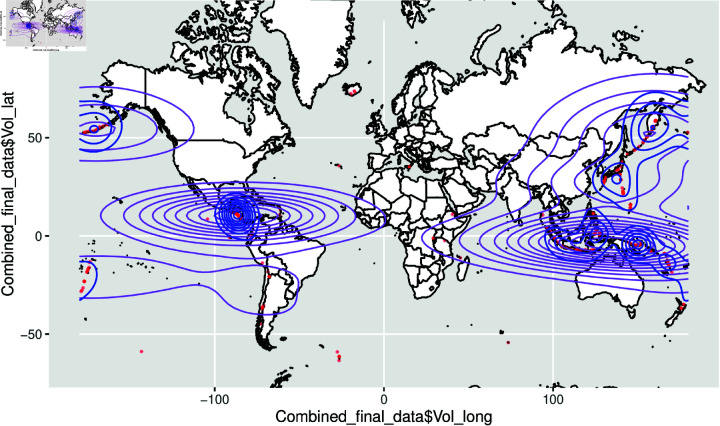
Plot of the volcano part of the dataset all over the two-dimensional world map with the densities.

**Fig 9:**
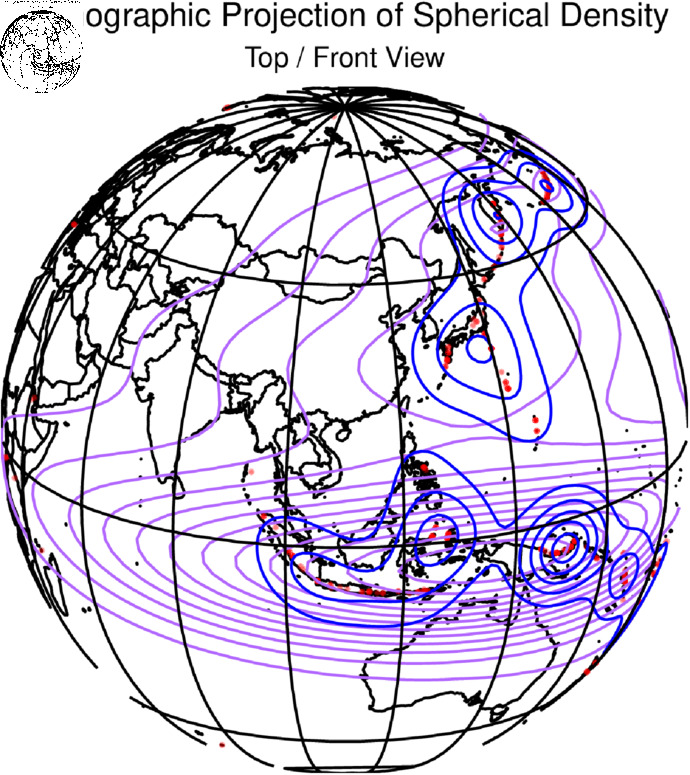
Plot of the volcano part of the dataset all over the three-dimensional world map with the density.

**Fig 10:**
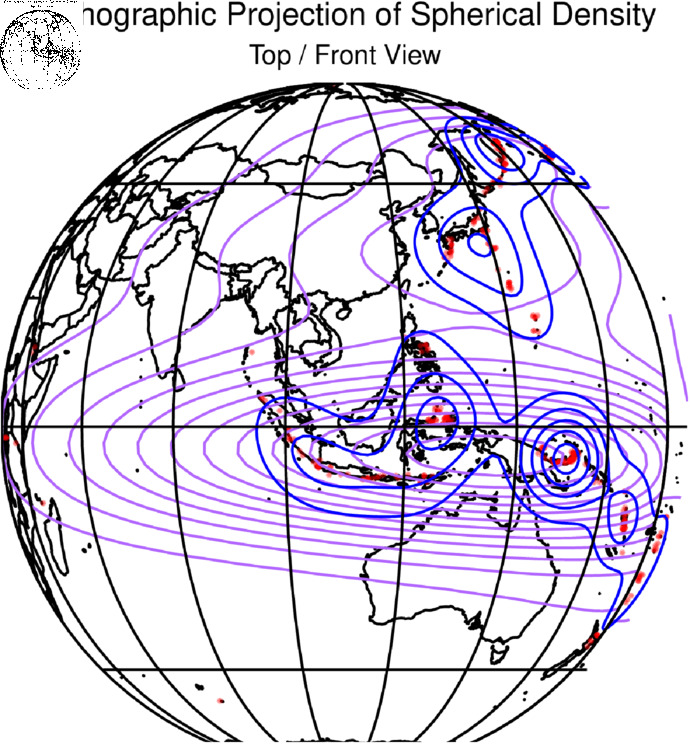
Plot of the earthquake part of the dataset all over the spherical world map with the densities.

**Fig 11:**
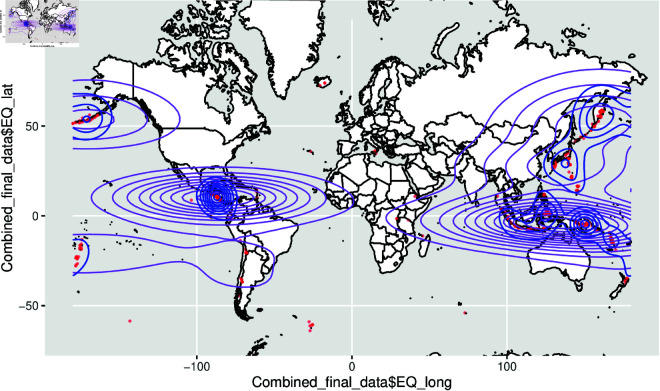
Plot of the earthquake part of the dataset all over the two-dimensional world map with the densities.

The spatial density plot given in Figs 8 and 9 represents the volcano plots and Figs 10 and 11 represents the spatial density plot of the Earthquake.

**Table 6:** Table containing the Bayesian Information Criteria Score for Each K value. From that, 14 partitions can provide a mixture of Fisher Von Mises Distribution for the location parameter(Longitude and Latitude together). Then checked 20 values of k from 1 to 20, where the optimal number of partitions is 14.

K Values	BIC	K Values	BIC
1	–54.06857	11	–1804.32167
2	–1114.07092	12	–1894.01874
3	–1268.51948	13	–1828.33372
4	–1424.21191	14	–1903.50719
5	–1467.47741	15	–1844.48016
y 6	–1507.52284	16	–1787.84740
7	–1730.72499	17	–1719.25160
8	–1748.07882	18	–1755.34958
9	–1799.561584	19	–1740.73555
10	–1815.63021	20	–1690.66637

**Table 7:** Table containing the Bayesian Information Criteria Score for Each K value. From that, 14 partitions can provide a mixture of Fisher Von Mises Distribution for the location parameter(Longitude and Latitude together). Checked 20 values of k from 1 to 20, and the optimal number of partitions is 14. Then, used the earthquake part of the combined dataset to get this table.

K Values	BIC	K Values	BIC
1	–49.87195	11	–1808.27896
2	–1112.30709	12	–1796.88277
3	–1261.87566	13	–1802.01574
4	–1417.94000	14	–1802.40150
5	–1505.86819	15	–1782.84385
6	–1501.74819	16	–1774.68752
7	–1731.04052	17	–1767.89547
8	–1758.54639	18	–1756.11820
9	–1778.91169	19	–1717.64136
10	–1789.60595	20	–1768.21031

### 4.6 Partitions for the volcano part dataset

Started with 20 of the partitions for the volcano part dataset; the details are in Table 6; obtaining an optimal 14 partitions can provide a mixture of Fisher Von Mises distribution for the location parameter (Longitude and Latitude together). Then, checked 20 values of k from 1 to 20, where the optimal number of partitions is 14. Then used the Volcano Part dataset to get this table. Then Get that this will give the weights (*ϕ*) or *α* values as 0.014515901, 0.078723287, 0.037609859, 0.004643843, 0.351158355, 0.01908429, 0.177467094, 0.062865949, 0.036460001, 0.041481650, 0.071710780, 0.070833349, 0.020899085, 0.012546552. This is done for the Volcano part of the dataset.

### 4.7 Partitions for the earthquake part dataset

Started with 20 of the partitions for the earthquake part of the combined dataset, and the details are given in Table 7. Checked values of k from 1 to 20, and the optimal number of partitions is 14, providing a mixture of Fisher von Mises Distribution for the location parameter (Longitude and Latitude together). The earthquake part of the combined dataset to get this table is used. Then it will give the weights (*ϕ*) or *α* values as 0.06643275, 0.02084306, 0.18108015, 0.06747089, 0.01665956, 0.00550244, 0.10448460, 0.01455958, 0.03741776, 0.01246680, 0.35071396, 0.01846428, 0.02948653, 0.07441763.

**Remark 6.** *It is observed that Earthquakes and volcanoes have the same 14 partitions as the optimal number of mixtures in the von Mises Fishers mixture model. In Table 5, It is observed that seven partitions are optimal for which it follow Von Mises distribution where, spherical to circular projection is taken, and in the spherical paradigm, it is 14 (Multiple of 7)*

## 5 Discussions

The primary findings of this work can be summarized in the following. This study identified distributional similarities between volcanic eruptions and earthquakes, supporting the geological explanations and relationships between these two natural phenomena.

The dataset constructed by collecting earthquake data within a 100 km radius of volcano eruption locations within one year. Count data for the number of earthquakes and volcano eruptions were used to assess the frequency of these events. It is proposed that empirical models and statistically validate the models based on that dataset. Although the model is empirical, it may provide a more subtle perspective on the potential interactions between volcanoes and earthquakes.

The study utilized 2D projection partitioning techniques, which resulted in seven partitions for the combined volcano and earthquake data. This partitioning suggests that distinct clusters or groups of events exhibit similar characteristics within the dataset. All partitions follow the vonMises distribution for both parts (volcano and earthquake), and the optimal number of partitions is 7.

The Von Mises Fisher distribution fit test was applied for the spherical analysis. This test indicated that the optimal number of partitions for volcanic eruptions was 14, while for earthquakes, it was 14. These results align with the 2D projection partitioning findings, further supporting the existence of distinct patterns within the data.

Previously some works are done. [45] presents a probabilistic framework using temporal hypergraphs to model and simulate multihazard interactions, aiding in civil infrastructure design, risk assessment, and disaster mitigation [46]. Recent studies have also explored the intricate relationships between volcanic activity and seismic events, providing insights that align with our findings. For instance, research by [47] and [48] has demonstrated the statistical correlation between pre-eruption seismicity and volcanic activity, reinforcing the significance of earthquake clustering near volcanic regions. Moreover, advancements in machine learning and probabilistic modeling, as highlighted by [49], have improved forecasting accuracy by integrating seismic, geodetic, and geochemical data. Studies such as [50] have further validated the role of spatial partitioning techniques in identifying seismic precursors to eruptions, supporting our approach using 2D projection partitioning and von Mises Fisher distribution. By incorporating these contemporary findings, our study contributes to the growing body of research that seeks to enhance the predictive capabilities of volcano-earthquake interactions.

### Practical implication

The probabilistic analysis presented in this study has significant implications for volcanic and seismic risk assessment, particularly in improving hazard forecasting and mitigation strategies. By identifying distributional similarities between volcanic eruptions and earthquakes, our approach provides a quantitative framework for assessing the likelihood of seismic activity preceding volcanic events. This can enhance early warning systems by integrating probabilistic models with real-time monitoring data, enabling authorities to make informed decisions regarding evacuation protocols and resource allocation. Furthermore, the use of statistical partitioning techniques, such as the von Mises Fisher distribution and 2D projection partitioning, allows for the classification of high-risk regions based on historical and real-time event clustering. These insights can be incorporated into risk assessment models used by government agencies, geophysical institutes, and disaster management organizations to refine hazard maps and optimize emergency response plans. By transitioning from deterministic to probabilistic forecasting, this study contributes to a more comprehensive risk management approach that accounts for uncertainty and variability in volcanic and seismic activity.

## 6 Conclusion & future work

This study provides a foundational framework for understanding the statistical relationship between volcanic eruptions and earthquakes through directional statistical modeling. By analyzing event distributions and clustering patterns using 2D projection partitioning and the von Mises Fisher distribution, we identified distinct statistical similarities that support geological explanations of volcano-earthquake interactions. These findings align with our research objective of enhancing probabilistic modeling approaches for volcanic and seismic hazard assessment.

### Key findings

- **Statistical Similarities:** The study demonstrated that volcanic eruptions and earthquakes share distributional characteristics, reinforcing their geological relationship.
- **Partitioning and Clustering:** The application of 2D projection partitioning revealed distinct event clusters, suggesting systematic patterns in volcano-earthquake interactions.
- **Directional Statistical Approach:** The use of von Mises Fisher distribution in spherical analysis provided further validation of event groupings, enhancing the robustness of our classification method.
- **Empirical Model Validation:** While the model remains empirical, it offers a novel perspective on identifying potential interactions between seismic and volcanic events, contributing to hazard assessment models.

### Broader implications

- **Advancing Hazard Forecasting:** The study contributes to improving probabilistic forecasting of volcanic and seismic hazards, supporting early warning systems in high-risk regions.
- **Data-Driven Decision Making:** By integrating statistical modeling techniques with real-world event data, the findings provide a quantitative basis for enhancing disaster preparedness strategies.
- **Future Research Directions:**
  - Incorporating **spatiotemporal models** to capture evolving patterns of seismic and volcanic activity over time.
  - Expanding the dataset with **global seismic-volcanic event records** to refine the statistical approach.
  - Applying **Bayesian statistical frameworks** to further enhance predictive accuracy and risk assessment.
  - Investigating how **geophysical parameters** (e.g., magma chamber dynamics, tectonic stress) influence observed clustering patterns.

Using n directional statistical tools and integrating advanced spatial and temporal analysis, future research can refine hazard assessment strategies and deepen our understanding of the complex geophysical processes governing volcano-earthquake interactions. This study serves as an important step toward developing a more comprehensive, data-driven approach for risk assessment in seismically active regions.
